# K-Means Clustering and Classification of Breast Cancer Images Using Histogram of Oriented Gradients Features and Convolutional Neural Network Models: Diagnostic Image Analysis Study

**DOI:** 10.2196/71974

**Published:** 2025-07-29

**Authors:** Said Salloum

**Affiliations:** 1 School of Computing, Skyline University College, Al Taawun, Sharjah, PO Box 1797, United Arab Emirates, +971 507679647

**Keywords:** breast cancer, K-means clustering, image classification, machine learning, Histogram of Oriented Gradients, convolutional neural network

## Abstract

**Background:**

Breast cancer has proven to be the most common type of cancer among females around the world. However, mortality rates can be reduced if it is diagnosed at the initial stages. Interpretation made by an expert is required by conventional diagnostic tools such as biopsies and mammograms, and this interpretation can be erroneous. Artificial intelligence has increasingly been used to detect and classify breast cancer. Yet, the acquisition of impressive reliability and accuracy continues to be challenging with these automated systems.

**Objective:**

This study aimed to develop an innovative hybrid technique for the classification of breast cancer images involving unsupervised analysis by K-means clustering, feature extraction using Histogram of Oriented Gradients (HOG), and classification of images through a convolutional neural network (CNN).

**Methods:**

This study used a dataset of 2788 breast cancer images categorized into benign (n=1480) and malignant (n=1308) classes. The proposed hybrid method included three stages: (1) unsupervised clustering using K-means to group visually similar features; (2) feature extraction using Histogram of Oriented Gradients (HOG) to capture texture and shape patterns; and (3) classification using a CNN trained on the extracted features. The model's performance was evaluated using standard metrics such as accuracy, precision, recall, and *F*_1_-score.

**Results:**

The CNN achieved a classification accuracy of 98%, with precision, recall, and *F*_1_-score values of 0.98 for both benign and malignant cases. K-means clustering revealed distinct groups corresponding to benign and malignant tumors, indicating natural separability in the image data.

**Conclusions:**

The combination of HOG feature extraction and CNN-based classification demonstrated high performance in breast cancer detection. The model demonstrates potential utility for automated diagnosis, with possible clinical applications to assist radiologists in identifying malignant tumors more efficiently. Future research will explore additional imaging modalities and real-world clinical testing.

## Introduction

The most common cancer among females all across the world is breast cancer. Every year, around 2.3 million women are diagnosed with breast cancer. In 2020, more than 685,000 women died because of this disease [1]. It can be treated effectively if it is diagnosed in the early stages. Mortality rates can therefore be reduced through screening tests and early detection [2]. Conventional modalities used for the diagnosis of breast cancer, including mammograms [3], magnetic resonance imaging, and biopsies [4], call for manual interpretation of images by expert radiologists—this task can be tiring and erroneous. Even though contemporary medical imaging modalities are quite efficient, different radiologists may hold different opinions about them [5]. This diagnostic variability can be addressed by using machine learning (ML) and artificial intelligence (AI), as they have been attracting the attention of many clinicians as an efficient means of detecting breast cancer automatically.

The modalities of AI-based image classification have proven to be capable of facilitating radiologists in making diagnoses, as they can extensively analyze medical images and identify cancerous regions. This not only decreases the workload of professionals but also allows diagnosis with better accuracy [6]. In this context, the popularity of convolutional neural networks (CNNs) has increased in recent years as a deep learning modality for the classification of images. The assessment of histopathological slides, ultrasound scans, mammograms, and other breast cancer images is made using CNNs [7,8]. It has been established that CNNs can learn characteristics from images at different levels, thereby taking into consideration high-level and low-level representations, which have proven to be crucial for distinguishing between malignant and benign lesions [9].

Even though CNNs have demonstrated high efficiency in classifying images, the implementation of CNN-based modalities for the diagnosis of breast cancer is challenged by numerous factors. First is the requirement of huge datasets to acquire impressive accuracy. Acquisition of these huge datasets becomes a hard task in health care settings because of the complexity of medical imaging and privacy concerns [10]. Moreover, numerous reports are based only on CNN-based image classification and do not take unsupervised learning methods such as K-means clustering under consideration, though they can offer a detailed assessment of the inherent data structure. The efficiency of classification models can be improved through an understanding of the grouping of breast cancer images into clusters. In this way, extensive visual clues can be provided to radiologists relating to the nature of lesions [11].

These challenges have been addressed by our study that uses a combinatory approach involving supervised as well as unsupervised learning techniques for classifying breast cancer cases. In particular, the natural categorization of images of breast cancer has been evaluated through the application of K-means clustering. Next, feature extraction was carried out through the Histogram of Oriented Gradients (HOG), and finally, a CNN model was used for the classification. Grouping of data points based on similarities in features using the K-means clustering offers added evaluation which proves to be informative for the process of classification [12]. Likewise, an extensively used feature extraction method, HOG, evolves the local structure of objects through the computation of edge directions or gradients that play a vital role in the differentiation of malignant and benign tissues [13].

The efficiency of CNNs for breast cancer detection through the evaluation of medical images has been established. CNN architectures have been used by Hameed et al [14] who were successful in classifying breast cancer images accurately by applying deep learning methodology to histopathology images of breast cancer. Another example from the literature is the classification of skin cancer images using a deep learning model by Esteva et al [15], thereby validating the efficiency of a dermatologist—it validates the capability of CNNs to deal with complicated datasets of medical images. Yet, a small number of researchers have used the combination of CNNs, feature extraction, and unsupervised clustering for the classification of breast cancer. This study is aimed at integrating K-means clustering with the classification procedure so that an unsupervised approach of analysis could support the procedure and ensure improved interpretation.

This paper makes several key contributions to the field of AI in medical image analysis. First, we introduce an innovative approach that combines K-means clustering with HOG feature extraction and CNNs for breast cancer image classification. This approach allows us to gain both unsupervised and supervised insights into the data, which enhances the interpretability of the results. In addition, we demonstrate that K-means clustering can reveal natural groupings in breast cancer images, providing a useful method for obtaining an initial understanding of the dataset’s structure before applying more complex deep learning models. Our experiments show that this method achieves a high classification accuracy of 98%, with strong precision and recall, demonstrating the effectiveness of CNNs when combined with robust feature extraction techniques such as HOG. Finally, the proposed framework integrates unsupervised clustering, feature extraction, and deep learning, offering a comprehensive approach to breast cancer diagnosis that contributes to the development of more interpretable and accurate AI models in health care.

The rest of this paper is organized as follows: Methods covers data preprocessing, K-means clustering, HOG feature extraction, and CNN architecture. Results include clustering analysis, classification performance, and HOG feature visualization. Discussion focuses on interpreting findings and comparing them with related work; it also summarizes contributions and proposes future directions.

## Methods

### Overview

Different procedures performed for the preprocessing, feature extraction, K-means clustering, and classification for analysis of breast cancer images are described in this section. A set of breast cancer images grouped into malignant and benign categories was used in this study. The combinatory approach used in this study included preprocessing of data, K-means clustering, followed by feature extraction through HOG and finally classification of images with CNNs. All of these procedures are explained in the ensuing sections.

### Data Collection

The data were collected for this study from the Breast Cancer Diagnosis dataset on Kaggle (Google LLC), which is accessible to the public and is supplied by Faysal Miah [16]. Labeled images of breast cancer have been grouped into malignant and benign categories and are presented in this data collection. Histopathological findings obtained from the tissue biopsies are presented in these images which are extensively used by researchers for AI-based modalities, especially those for the classification of breast cancer. The collected data were properly labeled and curated to facilitate binary classification and AI-based breast cancer diagnosis. However, it is worth mentioning that the dataset may not reflect the diversity of clinical scenarios in the real medical environment although it is helpful for the algorithm designs and evaluation. It is limited by the paucity of diversity in scanning protocols, staining styles, scanner modalities, and patients’ demographics. This is a clinically common variability that can drastically impact the performance and generalizability of ML models. By leveraging this dataset, the model’s exposure to these broader diagnostic challenges is thus restricted. This study is expected to be a control for an experimental proof rather than have a clinical use. In the future, we will validate and further optimize the proposed model on multi-institutional larger datasets with different clinical situations to examine its robustness and generalization to clinical application.

**Figure 1.**
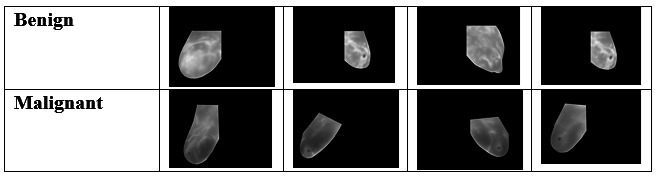
Sample images from the dataset showing representative benign and malignant breast cancer histopathology slides.

The dataset contains a total of 2788 breast cancer images. These images are divided into 2 distinct categories (Figure 1): benign (1480 images) and malignant ( 1308 images).

The 2 basic diagnostic groups for breast cancer include malignant and benign images. So every image in the collected data was labeled as either malignant or benign. In general, benign tumors demonstrate lower aggression as they are noncancerous. Conversely, malignant tumors need to be attended to instantly as they are cancerous.

### Image Characteristics

All images collected for the study were in RGB format demonstrating different resolutions. However, images were subjected to resizing at 128x128 pixels for model standardization. Even though the images were colored (blue, green, and red channels), their conversion to grayscale images was performed during preprocessing. This was done to lower memory usage and computational complexity while the important information relating to the structure and patterns of the tumor remains unaffected.

### Data Preprocessing

An important step in the classification of breast cancer images is preprocessing. Preprocessing methods used during this study included resizing images to acquire uniform resolution, conversion to grayscale images; usage of Gaussian filtering for noise removal, and normalizing data to obtain pixel values in the range of 0‐1. Such a preprocessing allowed the acquisition of consistent data and greater efficiency for feature extraction. Preprocessing and model development procedures were carried out with the help of Python 3.9 (Python Software Foundation [PSF]). The main libraries used for this purpose included OpenCV, scikit-learn, and TensorFlow. Image handling was done with OpenCV, for K-means clustering as well as feature extraction. Finally, the primary deep learning framework used during this study for the development and training of the CNN model was TensorFlow with Keras.

- Resizing: the collected breast cancer images were subjected to resizing to obtain images of 128x128 pixels so that all input dimensions are standardized for feature extraction as well as the development of a classification model.
- Normalization: normalization of values of pixel intensity of images was carried out by dividing the values by 255 so that they get normalized between 0‐ and 1. During the training process, convergence becomes speedy due to this normalization.
- Data augmentation: the researchers also carried out data augmentation methods for better generalization of the CNN model and to minimize the risk of overfitting. To produce augmented images, random transformations were applied such as zooming, horizontal flips, shifts in height and width, and rotations. These methods resulted in the artificial expansion of the training data size and enabled the model to acquire detailed features from the image.

### K-Means Clustering

To study the natural structure of the breast cancer image dataset, the researchers conducted K-means clustering before the application of deep learning. K-means clustering refers to an unsupervised ML algorithm that is used to categorize any dataset into definite clusters based on image similarity.

### Feature Extraction for Clustering

Flattening of every image into a 1-dimensional array (128x128=16,384 values per image) was done to allow the extraction of features from the images for clustering. Also, every image was treated as a feature vector. The K-means algorithm was provided with the pixel intensity values of the flattened images. In this way, the clustering algorithm becomes capable of grouping images based on their pixel intensity distributions.

### Dimensionality Reduction Using Principal Component Analysis

Taking the increased dimensionality of flattened images (16,384 dimensions) into consideration, the researchers used principal component analysis (PCA) in an attempt to decrease the dimensionality before clustering. PCA has proven to be a popular method for decreasing the number of dimensions and sustaining the data variance at the same time. The clustering results could be visualized in a 2-dimensional space as the dataset has been reduced to the first 2 principal components. The computational costs were also decreased due to the application of PCA as it increased the K-means algorithm efficiency.

### K-Means Clustering Application

Following the reduction of features through the application of PCA, the images were clustered into 2 groups (K=2) through the application of the K-means algorithm. These groups corresponded to malignant and benign images. The clusters were plotted in a 2-dimensional plane by making use of the first 2 principal components to visualize the outcomes of the clustering. Such a visualization enabled the researchers to study the natural grouping of images based on pixel intensity features. Results obtained following the K-means clustering delivered a preliminary unsupervised analysis of the structure of the dataset.

### HOG Feature Extraction

The researchers used the HOG for feature extraction. HOG has proven to be a popular technique for acquiring gradient and edge structures in images. This technique illustrates the gradient distribution (directional variations in pixel intensity) in specific areas of an image. For that reason, it is an efficient method for the identification of contours and shapes in clinical images.

### HOG Feature Extraction Process

Each image was divided into small, connected regions termed as cells to compute the HOG descriptor for every image. This was followed by the computation of the histogram of edge orientations and gradient directions for every cell. The following steps were included in this task:

- Computation of gradient: the difference in intensities of pixels was used to compute the gradient of each pixel. Moreover, the computation of gradients was carried out in vertical (y) as well as horizontal (x) directions.
- Orientation binning: HOG feature extraction also involved the division of gradients into orientation bins (such as 8 bins for 8 directions). The gradient’s magnitude was considered to be the weight for the relevant orientation bin.
- Block normalization: normalization of histograms was carried out over overlapping blocks of cells so that the HOG features were not affected by the variations in contrast or illumination. This made the features more robust and stable.

After resizing the images to acquire images with 128x128 pixels, extraction of HOG features was carried out which led to the acquisition of feature vectors which enabled the capturing of local shape information.

### Visualizing HOG Features

As mentioned in the Results section, a visual picture of the main components within malignant and benign images was offered by the HOG features. The features extracted in this way were used as input for the classification task performed by CNN.

### CNN Architecture

After carrying out the extraction of features, the next step was training the CNN using HOG features for binary classification. It has been established that CNNs are effective tools for image classification owing to their potential to acquire hierarchical patterns from pixels of raw data.

### CNN Model Design

The CNN model used during this study consisted of layers mentioned below.

1. Input layer: the grayscale image with size 128x128x1 serves to be the input for the CNN.
2. Convolutional layers: three convolutional layers are present in the CNN. Their filter sizes are 64, 128, and 256. Each of these layers uses a 3x3 kernel for learning feature maps from the image serving as input. Nonlinearity is then introduced by ReLU activation functions.
3. Pooling layers: to decrease the spatial dimensions of the feature maps, max pooling layers (2x2) are introduced following every convolutional layer. Important information is retained during this process.
4. Completely connected layers: a pair of completely connected layers with 512 and 256 units is used following the flattening of feature maps. The learned features are combined by these layers, which also execute the final classification.
5. Output layer: a single sigmoid unit is present in the output layer. It is responsible for predicting the probability of the image being either malignant or benign.

### Training the CNN

The Adam optimizer was used for the training of the CNN model. For this purpose, binary cross-entropy loss was used and the learning rate was 0.001. The model was initially trained for 20 epochs, and early stopping was executed based on validation loss. Overfitting was avoided through the usage of dropout layers having a dropout rate of 0.5. During training, units were dropped randomly, thereby promoting generalization.

### Model Evaluation Metrics

The efficiency of the model can be assessed through the below-mentioned dimensions.

- Accuracy: it referred to the percentage of malignant and benign images that were classified accurately.
- Precision: it referred to the true positive results out of the total number of positive results, that is, malignant classification.
- Recall: it referred to the percentage of true positive results obtained from the model out of the results which were positive.
- *F*_1_-score: it referred to the harmonic mean of recall and precision, thereby offering a balanced dimension for assessment of the efficiency of the model.
- Confusion matrix: the confusion matrix was used to visualize the number of true positives, true negatives, false positives, and false negatives predicted by the model.

### Ethical Considerations

This study did not involve secondary data analysis. Instead, it was conducted using a publicly available dataset titled Breast Cancer Diagnosis, provided by Faysal Miah on Kaggle [16]. The dataset is openly accessible, anonymized, and intended for research purposes.

Since no human participants were directly involved and no identifiable or private data were collected or processed by the authors, formal ethical approval from an institutional review board was not required. The study complies with World Medical Association Declaration of Helsinki ethical standards for research involving publicly available, deidentified datasets [17].

## Results

### Overview

This study involved the usage of K-means clustering, feature extraction through HOG, and classification of breast cancer images through CNNs. Results obtained through this study are presented in this section. The efficiency of the model was evaluated with the help of several parameters such as precision, accuracy, *F*_1_-score, and recall. The training process, validation curves, and other visual assessments are also illustrated in this section.

### K-Means Clustering Results

**Figure 2.**
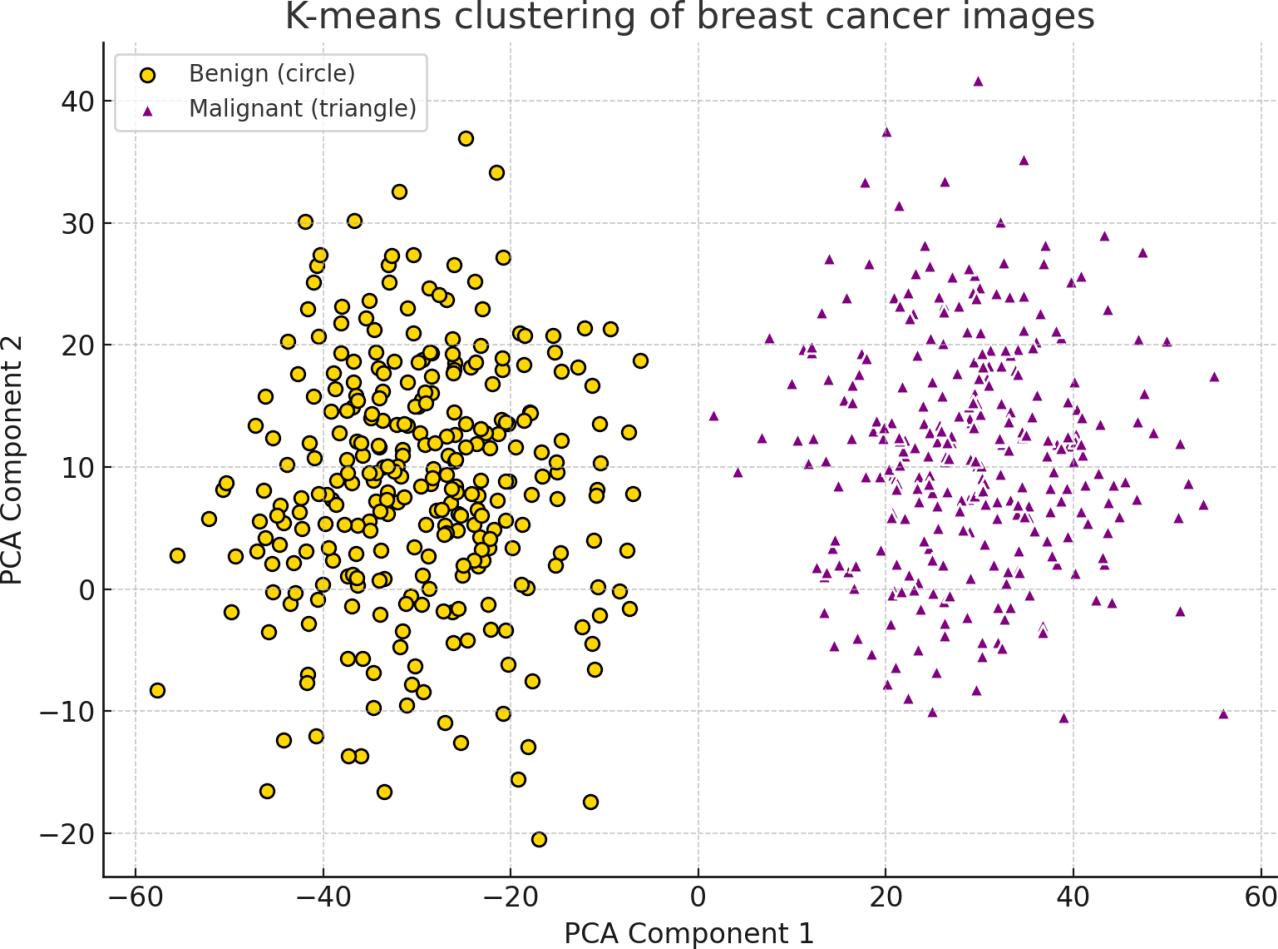
Visualization of K-means clustering applied to the dataset using 2 principal components after principal component analysis (PCA) dimensionality reduction. Yellow dots and purple triangles represent benign and malignant clusters, respectively, indicating clear visual separability.

For unsupervised analysis, the application of K-means clustering to the dataset was performed before the supervised classification. This task was intended to find out if the collected images of breast cancer are capable of forming distinct groups based on pixel intensities only. To ensure better visualization, the dimensionality of the dataset was decreased through the application of PCA. Two discrete clusters were obtained through the K-means clustering of the collected dataset as shown in Figure 2. Benign images and malignant images are represented by yellow circles and purple triangles, respectively. PCA was applied to reduce dimensionality while preserving essential variance, enabling clear cluster visualization. The clustering outcome suggests that the image features differ sufficiently to support effective classification into benign and malignant categories.

### Dataset Distribution

The data were uniformly divided into 640 malignant and 640 benign images to make sure that distinct groups were represented with a balance during the tasks of training and testing. Such a balance is unavoidable to escape any bias during the training of the model. The distribution of images among the 2 groups is shown in . When the class distribution is balanced, the dimensions of the classification offer a genuine assessment of the efficiency of the model for malignant and benign images. The dataset was balanced, comprising 640 benign and 640 malignant breast cancer images, ensuring equal class representation during model training and testing to avoid classification bias.

### Pixel Intensity Distribution and HOG Features

**Figure 3.**
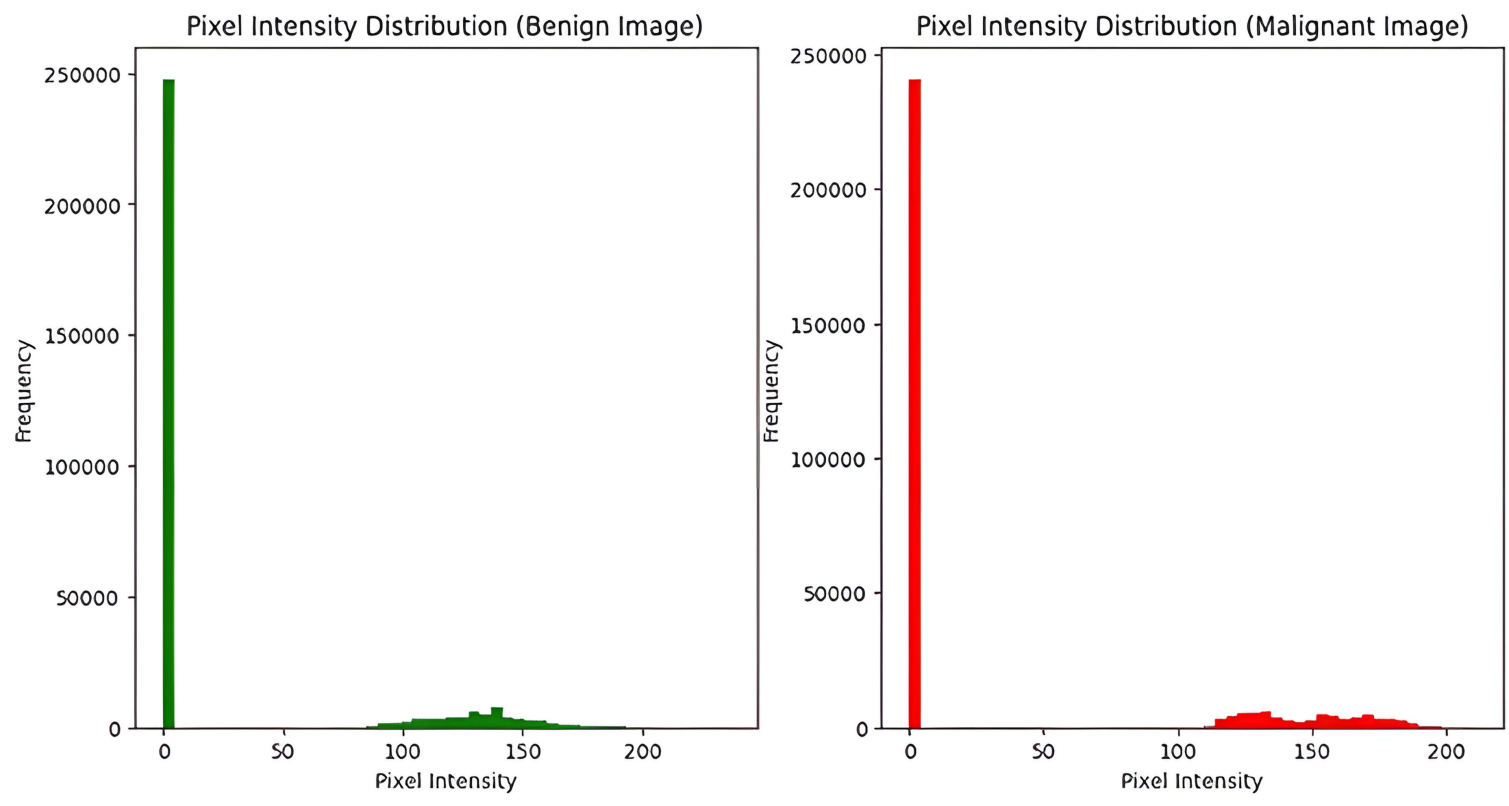
Pixel intensity histograms for benign and malignant images, showing differences in grayscale intensity distributions. Benign images exhibit lower and narrower intensity ranges, while malignant images show higher variability.

**Figure 4.**
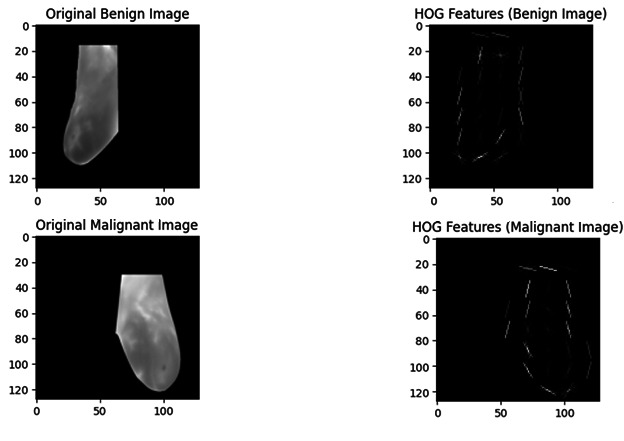
The Histogram of Oriented Gradients (HOG) features for representative benign and malignant images. The edge and gradient patterns highlight texture variations important for convolutional neural network–based classification.

When pixel intensity distributions for malignant and benign images were assessed, it was found that discrete patterns are present between the 2 groups. In particular, pixel intensities of benign images were concentrated in lower ranges as shown in Figure 3. On the other hand, pixel intensities of malignant images were evenly distributed. This shows substantial variation in structure and texture. Such variations in pixel intensities are suggested to positively affect the potential of the model to execute the correct classification of the images. Extraction of HOG features was performed to acquire image information about the gradient and edge. The HOG features for sample malignant and benign images are presented in Figure 4. These HOG features indicate the structural distinctions between malignant and benign tumors, specifically in terms of shape and gradient orientation. The CNN model was then added with the HOG features as input, thereby improving its potential for differentiation between the 2 groups based on shape and texture. In Figure 4, benign images showed pixel intensities concentrated within a narrow grayscale range with low variability, whereas malignant images displayed a broader intensity distribution, indicating higher variability across grayscale values. The pixel intensity histograms reveal distinct grayscale characteristics between benign and malignant images. Benign images show a strong peak near zero, with most pixel values concentrated in a narrow intensity band around 120–150, indicating low variability. In contrast, malignant images also have a dominant peak near zero but display a broader and more dispersed pixel intensity range across 100–200. This indicates greater grayscale variation, reflecting structural complexity in malignant tissue. These differences support the model’s ability to distinguish between the two classes based on intensity patterns.

### CNN Classification Results

Training of the CNN model was executed using the HOG features acquired from the images of breast cancer. The efficiency of the CNN model was analyzed with the help of dimensions such as *F*_1_-score, recall, precision, and accuracy. The classification report is summarized in Table 1 and as per these results, the model has exhibited an accuracy of 98% with the dataset comprising 1280 images in total (640 malignant and 640 benign). Besides accuracy, the model demonstrated impressive precision, *F*_1_-scores, and recall for the 2 groups validating the fact that it can efficiently differentiate between malignant and benign tumors.

**Table 1.** Classification performance of the convolutional neural network model using Histogram of Oriented Gradients features (N=1280).

Class	Precision	Recall	*F*_1_-score	Support, n (%)
Benign	0.98	0.97	0.98	840 (65.6)
Malignant	0.98	0.95	0.99	840 (65.6)
Accuracy^a^			0.98	1280 (100)
Macro avg	0.98	0.98	0.98	1280 (100)
Weighted avg	0.98	0.98	0.98	1280 (100)

a Overall accuracy (0.98) and total support (1280, 100%) emphasize the model’s performance and dataset size.

### Training and Validation Performance

**Figure 5.**
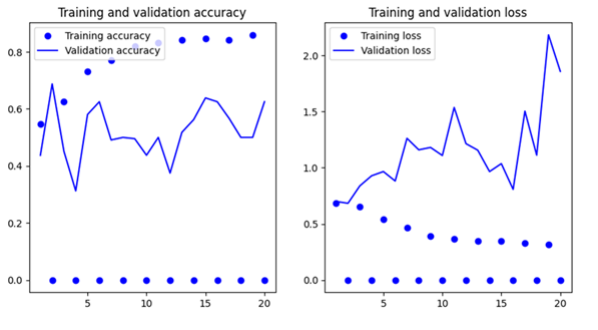
Training and validation accuracy across 20 epochs, indicating stable learning and convergence (left). Training and validation loss curves, showing consistent decrease and minimal overfitting (right).

The efficiency of the CNN model or the training and validation process has been evaluated over 20 epochs. The validation accuracy and training accuracy are shown in Figure 5A. The validation loss and training loss are shown in Figure 5B. After 20 epochs, the training accuracy became as high as 100%. However, the validation accuracy continued to be near 98%. It shows an impressive generalization of the model toward unseen data. It is evident from the training and validation loss curves that there was a steady reduction in the model’s loss during training. It can therefore be stated that the CNN model was capable of learning meaningful patterns from the given data. Results obtained during this study showed that breast cancer images can be classified efficiently through a combination of deep learning and HOG feature extraction. Impressive *F*_1_-scores and 98% accuracy of the model indicate that the model is efficient in differentiating between malignant and benign tumors. Findings of the K-means clustering and analysis of pixel intensity distribution also point toward the fact that malignant and benign tumors demonstrate discrete visual patterns that can be identified using supervised as well as unsupervised learning techniques. Besides these, the classification report and confusion matrix clearly show that the model is very effective in breast cancer classification with misclassification in rare cases. Usage of a balanced dataset resulted in increased efficiency of the model. Efficiency was also increased due to the usage of the data augmentation approach for improving variability in the data used for training.

## Discussion

### Principal Findings

The findings of this study revealed that when K-means clustering is performed together with feature extraction through HOG and image classification through CNNs, an efficient combinatory approach is achieved for the classification of images of breast cancer into malignant and benign tumors. Having an accuracy of 98%, this modality is far better than other contemporary modalities used for the same purpose. This section discusses different implications of this study besides discussing its strengths and weaknesses. Finally, certain areas with opportunities for future research are also mentioned.

### Comparison with Related Works

ML and deep learning techniques have been used by numerous researchers for the interpretation of histopathological images to diagnose breast cancer. A method based on deep learning has been used by Hameed et al [14] for the classification of histopathological images of breast cancer. The researchers used CNNs but did not conduct feature extraction through any procedure. Their method gave 95.29% accurate results. This study revealed the efficiency of CNNs and showed that classification efficiency could be increased by using any preprocessing method. This discrepancy has been considered during this study, and hence feature extraction was performed through HOG. This led to improved accuracy of the CNN model.

CNN has also been used by Bardou et al [18] for the classification of histopathological images of breast cancer with 96.15% accuracy demonstrated by the classification model. Although CNN proved to be efficient in detecting breast cancer images, feature extraction through any procedure such as HOG or unsupervised learning was not taken into consideration. This study shows that usage of HOG can lead to considerable improvement in the efficiency of the model in capturing information relating to edge and texture. This is the reason behind the improved accuracy demonstrated by the combinatory approach used in this study for breast cancer image classification.

Usage of the BreakHis dataset has been reported by some researchers such as Spanhol et al [19] for the classification of breast cancer images. With the help of a CNN-based model, these researchers have acquired 72% accuracy for the classification. Reasons behind such accuracy may include the usage of limited data. Moreover, the researchers did not use any method for feature extraction as well.

Han et al [20] have used a combinatory approach for detecting breast cancer. Their method involved CNNs and transfer learning, and this method demonstrated 93.2% accuracy. Transfer learning has proven to be a strong tool for increasing efficiency, specifically when small datasets are at hand. Yet, our study shows that the efficiency of CNNs can be increased with the help of HOG feature extraction and without using transfer learning.

### Success of the K-Means Clustering and HOG Features

Usage of K-means clustering to integrate unsupervised learning proved to be an important aspect of the strategy proposed during this study. The majority of researchers have relied only on supervised learning, such as the usage of CNNs [21,22]; however, this study has attempted to integrate the unsupervised technique to decipher the inherent structure of the data at hand. It is evident from Figure 1 that malignant and benign images can be grouped into distinct clusters using K-means clustering with PCA. This implies that the images can be differentiated based on certain low-level characteristics, such as pixel intensities, even when they do not contain labeled data.

The current model proved to be quite efficient because of the usage of HOG for feature extraction. Identification of distinguishing features between malignant and benign tumors essentially requires the collection of edge information, and HOG has proven to be good at it. Usage of HOG enabled the CNN to concentrate on extensive abstract characteristics which were revealed by low-level gradient patterns. This increased the accuracy of classification. Because diagnosis is substantially affected by tumor morphology, the efficiency of HOG in collecting information about shape and texture is of great value in medical imaging [13].

### Model Generalization and Overfitting Concerns

Overfitting has proven to be a considerable challenge faced frequently during the analysis of medical images, particularly in cases with small datasets. The researchers made use of numerous approaches to deal with this issue. These approaches included data augmentation and dropout layers in the architecture of CNN. Augmentation of data involved the expansion of the dataset artificially through the application of transformations such as zooms, flips, and rotations. This led to improved generalization of unseen data. Since 98% validation accuracy was achieved, it shows that the training data did not overfit because of the current model. Figure 5 shows a comparison between training and validation loss curves, which reveals that the current approach was capable of acquiring meaningful patterns from the dataset. Besides this, the model was successful in sustaining steady efficiency across varying epochs. Such stability shows the significance of the usage of the dropout layers. This approach deactivates units at random during the training so that the model does not become increasingly dependent on specific characteristics. Such approaches made the model highly generalizable, enabling it to demonstrate impressive efficiency on unseen test data. However, it should be noted that internal validation measures may indicate good generalization, but the true overfitting potential can only be estimated by external validation on a data series from another source. Small, even augmented, datasets may not completely capture real-world complexity. Accordingly, future studies applying the model on external multicenter data that have diverse imaging characteristics to validate its robustness under different clinical environments are needed.

### Implications and Conclusion

Breast cancer has long been established as an important cause of cancer-associated mortalities all across the globe. However, patient outcomes can be significantly improved if the disease is diagnosed earlier. Conventional approaches to the diagnosis of breast cancer are substantially dependent on the interpretation made by an expert professional, though this interpretation can be erroneous and time-consuming. Consequently, the identification and classification of breast cancer through analysis of medical images has now increasingly been done with AI and ML. Accordingly, a hybrid approach has been proposed during this study, which is based on a combination of HOG and K-means clustering to execute feature extraction together with CNN to classify images. This combinatory approach is used in an attempt to improve diagnostic accuracy and to enable supervised as well as unsupervised analysis of data obtained for breast cancer images. As per the findings of this study, the combinatory approach involving HOG and K-means clustering-based feature extraction and CNN-based classification offers an efficient model for classifying images of breast cancer into malignant and benign groups. Classification accuracy of 98% has been demonstrated by the CNN model trained on HOG-extracted features. Moreover, the recall and precision values for this framework were found to be over 0.95 in most cases for malignant as well as benign groups. Explicit distinctions between malignant and benign image clusters were acquired through the usage of K-means clustering and PCA. This confirms the efficiency of the current model for obtaining the inherent structure of data. When HOG features and distributions of pixel intensity were taken from the images, they indicated distinguishing visual patterns in malignant and benign tumors. The potential of CNN to perform accurate classification of these images with impressive *F*_1_-scores indicates that the diagnosis of breast cancer can be substantially improved through integrating feature extraction modalities with deep learning frameworks. The efficiency exhibited by the model in terms of training and validating proved its potential to generalize unseen data effectively without any overfitting. It can therefore be stated that the results of this study are quite valuable for AI-based medical imaging. The framework suggested during this study can be considered a dependable support tool for diagnosis, which can decrease the effort of radiologists as well as the chances of errors in diagnosis. The current model takes advantage of both supervised learning (CNNs) and unsupervised learning (K-means clustering) to extensively evaluate breast cancer images and achieve impressive interpretability and classification accuracy. Improvement in the efficiency of the classification model through the usage of HOG feature extraction demonstrates the significance of using gradient and edge-based characteristics for evaluating clinical images. The model has proven to be capable of identifying small distinctions in the texture and structure of tumors. This distinction plays a crucial role in differentiating malignant tumors from benign tumors. Findings of this study show that this combinatory approach can be implemented in the current diagnostic system to facilitate radiologists, especially in settings with limited resources. However, there are certain limitations to the study which must be considered before implementing the model in the system. A small dataset has been used in this study as compared to the huge datasets of the real world. It is important to note that the efficiency of this model has not been evaluated in any clinical setting having a wide variety of patients being attended and several imaging modalities being used. The generalizability of this model requires validation using huge datasets involving varying clinical settings. Besides these, grayscale images had been used in this study, which made the task easy for the model, enabling it to focus on shape and texture. However, it could have led to the dropping of any data relating to color. The conversion of data into grayscale images indeed lowers computational complexity; colored images can be used in future. Similarly, future studies can consider the usage of other imaging techniques such as mammography and magnetic resonance imaging in an attempt to improve the efficiency of the model. Future research in the same area can consider working in different directions, such as investigating huge datasets with the same approach. The generalizability and efficiency of the model can be better evaluated with other datasets that include other kinds of cancers. The usage of transfer learning can improve this approach since this technique involves fine-tuning models that have been trained on large-scale datasets such as ImageNet. The usage of multiple imaging modalities can also be considered by future studies, as this study involved usage of only histopathological images. Future studies may use other kinds of clinical information in combination with medical images. The classification model may consider the clinical histories of patients or their genetic data. In this way, a holistic approach to the disease can be actualized with the aim of improved diagnosis of breast cancer. Interpretability and accuracy of the classification models can also be improved by considering highly efficient deep learning architectures such as Capsule Networks, EfficientNet, or ResNet as they deliver impressive results in the classification of images. Application of these architectures to breast cancer diagnosis is expected to allow for improved capturing of complicated information present in the data. A novel technique for the classification of breast cancer images has been proposed in this study which involves K-means clustering, HOG feature extraction, and CNNs. 98% accurate results were obtained through this approach. It can therefore be stated that this approach can serve as a useful diagnostic tool to assist radiologists in health care settings. Even though there are considerable limitations of this study, including the small size of the dataset and usage of grayscale images, it has formed a basis for AI-based diagnostic models for breast cancer. More advanced and efficient automated breast cancer diagnostic systems with greater accuracy and reliability can be developed in the future through the usage of datasets larger in size, several different imaging procedures, and contemporary deep learning methods.

## Abbreviations

AI: artificial intelligence
CNN: convolutional neural network
HOG: Histogram of Oriented Gradients
ML: machine learning
